# Accelerated Free Energy Estimation in *Ab Initio* Path Integral Monte Carlo Simulations

**DOI:** 10.1021/acs.jpclett.5c02193

**Published:** 2025-10-06

**Authors:** Pontus Svensson, Fotios Kalkavouras, Uwe Hernandez Acosta, Zhandos A. Moldabekov, Panagiotis Tolias, Jan Vorberger, Tobias Dornheim

**Affiliations:** † 682670 Center for Advanced Systems Understanding (CASUS), D-02826 Görlitz, Germany; ‡ 28414 Helmholtz-Zentrum Dresden-Rossendorf (HZDR), D-01328 Dresden, Germany; § Space and Plasma Physics, 7655 Royal Institute of Technology (KTH), Stockholm SE-100 44, Sweden

## Abstract

We present a methodology for accelerating the estimation of the free energy from path integral Monte Carlo simulations by considering an intermediate artificial reference system where interactions are inexpensive to evaluate numerically. Using the spherically averaged Ewald interaction as this intermediate reference system for the uniform electron gas, the interaction contribution for the free energy was evaluated up to 18 times faster than the Ewald-only method. Furthermore, an extrapolation technique with respect to the quantum statistics was tested and applied to alleviate the Fermion sign problem. Combining these two techniques enabled the evaluation of the free energy for a system of 1000 electrons, where both finite-size and statistical errors are below chemical accuracy. The general procedure can be applied to systems relevant for planetary and inertial confinement fusion modeling with low to moderate levels of quantum degeneracy.

## Graphical abstract

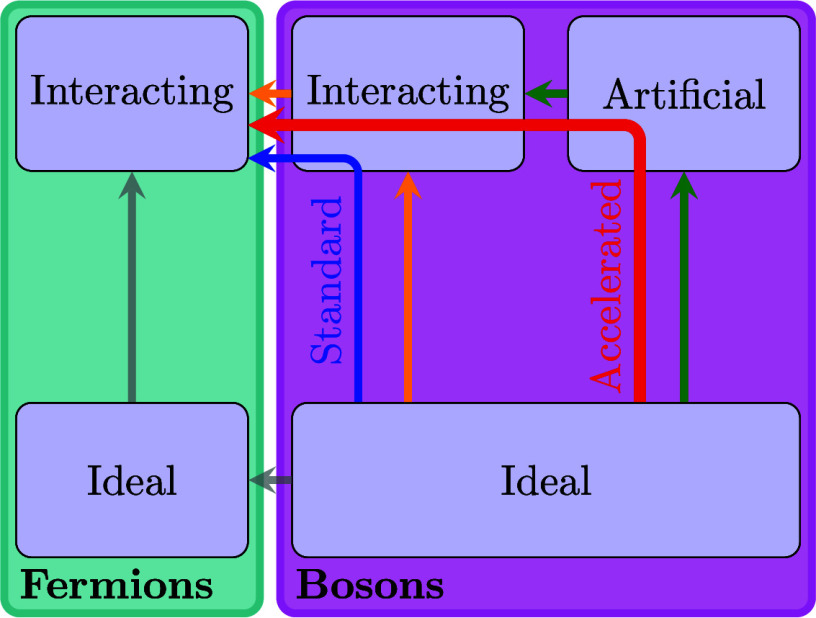

The description of thermal systems of interacting Fermions is a cornerstone of our understanding for a wide range of quantum systems, including ultracold atoms,^1,2^ quantum dots,^3,4^ and dense plasmas.^5^ In particular, dense quantum plasmas are abundant in astrophysics, where they are found in gas giants^6–8^ such as Jupiter,^9^ Saturn^10^ and some classes of exoplanets,^6^ and stars^11^ most notable in later stages of stellar evolution in the form of red giants,^12^ white dwarfs^13,14^ and the atmospheres of neutron stars.^15,16^ However, high-density plasmas are also central in human-made applications such as inertial confinement fusion (ICF)^17–19^ and the synthesis of novel materials.^20^ In recent groundbreaking experiments, ICF implosions have exceeded the Lawson criteria and achieved capsule gain,^21^ a key step toward achieving energy production through the ICF concept.

A formidable regime of dense plasmas to model theoretically is warm dense matter (WDM), which is characterized by a complex interplay between interactions, quantum degeneracy, and thermal excitations.^5,22,23^ All the previously mentioned effects must be taken into account as both *r*_*s*_ – the ratio between the Wigner-Seitz radius and the Bohr radius – and θ – the ratio of the thermal excitation energy and the electronic Fermi energy – are of order unity, which characterizes the strength of interactions and quantum degeneracy, respectively. Therefore, there remain uncertainties in the fundamental properties of WDM, such as the equation of state (EOS) and transport properties, which limit predictive modeling of, for example, the Jovian interior^8,24^ and ICF implosions.^25^

The most widely used description for WDM systems is a hybrid method (DFT-MD),^26^ where electrons are described using density functional theory (DFT),^27,28^ while ions are treated by molecular dynamics (MD).^29^ The fidelity of DFT strongly depends on the unknown exchange-correlation functional and practical calculations often resort to approximations based on the properties of the uniform electron gas (UEG).^30–32^ Path integral Monte Carlo (PIMC)^23,33,34^ provides suitable benchmark at finite temperature, since it is exact within the statistical error. However, for Fermionic systems, PIMC is limited by the Fermion sign problem (FSP) in the number of particles and the level of quantum degeneracy it can model.^35^ The FSP arises because all Fermionic observables are ratios where the denominator is the average sign *S*, which decreases exponentially with particle number and the inverse temperature.^35,36^ This vanishing sign causes computations of large or cold systems to be dominated by statistical errors.^37^

A large number of methods have been introduced to address the FSP, e.g., restricted PIMC,^2,38^ permutation blocking PIMC,^39^ configuration PIMC,^40^ Fermionic PIMC^41,42^ and methods based on the Wigner formulation of quantum mechanics,^43^ but no method is able to fully alleviate the FSP in the whole phase space without approximation. Here we consider the ξ-extrapolation method suggested by Xiong and Xiong,^44^ where an additional ξ parameter that continuously interpolates from the bosonic (ξ = 1) to the Fermionic (ξ = −1) limit is introduced. By introducing an empirical model for the ξ-dependence, calculations can be carried out in the FSP-free parameter regime and extrapolated to the Fermionic results, circumventing the exponential computational cost with respect to the particle number.^45,46^ The validity of the extrapolation method is limited to weak and moderate degenerate systems (θ ≥ 1.0) where the statistical attraction and repulsion for bosons and Fermions, respectively,^47^ are not dominant.^45^ The extrapolation will fail if the structural properties between the bosonic and Fermionic systems are too different, and more formal attempts to understand the ξ-dependence in the ground state have been made recently.^48^ However, the method has been successfully applied at moderate degeneracy for the computation of energy,^45,49,50^ static structure,^45,51–53^ imaginary time correlation functions,^45,53^ density response,^51,54^ and the average sign itself.^55^

The (Helmholtz) free energy is central for our understanding of thermal systems, for example it is directly related to the exchange-correlation functional in DFT^28,56,57^ where finite-temperature corrections are key at intermediate temperatures,^26,58–60^ but the free energy is also commonly used to investigate the stability of different phases.^61–63^ As the free energy is a thermodynamic potential, a free energy parametrization automatically yields a self-consistent EOS where all thermodynamic properties are obtained through differentiation. So far, first-principles tabulations of the EOS have focused on energy and pressure,^64^ but semiempirical constructions commonly model the free energy.^65,66^ By accelerating first-principles computations of the free energy, we are moving closer to reliable and internally consistent equation of state tables in the WDM regime.

In this letter, we present computations for the free energy of the spin-unpolarised UEG from PIMC with unprecedentedly large system sizes and low statistical errors. Using a combination of robust extrapolation techniques and the introduction of an intermediate reference system where interactions are computationally cheap, we are able to model *N* = 1000 electrons. The efficiency of this scheme allows us to evaluate the free energy to well within chemical accuracy (i.e., 1 kcal/mol ≈ 1.6 mHa^67^). In the main text, we focus on the condition *r*_*s*_ = 3.23 and θ = 1.0 characteristic of the electronic conditions possible to achieve in hydrogen jet experiments,^68–70^ but the methodology is general and applicable for either bosons and not too degenerate Fermi systems. The complete analysis for the UEG at *r*_*s*_ = 10 is given in the Supporting Information.

The partition function or the free energy is not a thermodynamical average *per se*, but relates to a volume in phase space.^71^ Therefore, the free energy is not readily available from an Monte Carlo (MC) or molecular dynamics (MD) simulation, and the thermodynamic integration (TI)^71,72^ method or the adiabatic connection (AC) formula^73^ has traditionally been used for its computation. Both methods require multiple computations, e.g., with an interaction that can be smoothly turned from that of a reference system—commonly the ideal system—to the target system. Moreover, the application of the AC method to inhomogeneous systems, such as the electronic problem in the external potential of a fixed ion configuration, poses an additional obstacle. Recently, Dornheim et al.^74^ introduced the extended ensemble technique in which the free energy differences between systems 1 and 2 can be directly computed.^74^ The extended partition function in question is

1 $$Z_{\mathrm{ext}}=cZ_{1}+Z_{2}$$

where *Z*_*i*_ is the partition function of system *i*, and *c* is an arbitrary coefficient that is chosen to optimize the ergodicity.^74^ In the extended ensemble, the difference in free energy per particle *f*_*i*_ between the two systems is directly related to the thermal averages in the extended ensemble ⟨·⟩_ext_ via

2 $$f_{1}-f_{2}=-\frac{k_{B}T}{N}\;\log\left(\frac{c^{-1}\langle {\mathrm{\delta ̂}}_{1}\;\rangle_{\mathrm{ext}}}{\langle {\mathrm{\delta ̂}}_{2}\;\rangle_{\mathrm{ext}}}\right)$$

where δ̂_*i*_ is one in system *i* and zero otherwise, *k*_B_ *T* is the temperature in energy units, and *N* is the number of particles.

The Hamiltonian *Ĥ*_η_ = *K̂* + *ηV̂* where *K̂* is the kinetic energy operator and *V̂* is the Ewald summation,^32,75^ interpolates between the ideal (η = 0) and interacting systems (η = 1). By considering η = 0 and η = 1 for the two systems in eq 2 along with exact results for noninteracting systems,^76,77^ the free energy for bosons can be computed^74,78^ in what we will refer to as the η-ensemble. However, for a large number of particles, it was found practically difficult to ergodically explore the entire extended ensemble due to the presence of configurations in the ideal system that are strongly suppressed in the interacting case.^78^ Therefore, multiple intermediate η-steps are introduced, a prevailing strategy in free energy calculations with substantially different configurational spaces.^71^ Structurally, the η-ensemble becomes reminiscent of the TI with the in-between steps. However, in the η-ensemble, η-values with a finite difference are considered, whereas in TI a continuous function of the coupling constant is integrated.

The η-ensemble is performed in the bosonic sector and is therefore FSP free, but a large number of intermediate η-steps will result in a prohibitive computational cost for accurate free energy calculations for large *N*. The majority of the computational cost in each MC step comes from the evaluation of the Ewald summation. To avoid this problem, we evaluate the η-ensemble using a nonphysical interaction or artificial interaction, *V̂* → *V̂*_art_, which is computationally cheap, and any error is corrected for in a second step henceforth referred to as the *a*-ensemble. The *a*-ensemble concerns the Hamiltonian

3 $${Ĥ}_{a}=\mathrm{K̂}+a\mathrm{V̂}+\left(1-a\right){\mathrm{V̂}}_{\mathrm{art}}$$

were *a* = 0 and *a* = 1 is used for the two systems in eq 2. If the physical interaction *V̂* and the artificial one *V̂*_art_ are sufficiently similar, no intermediate *a* steps are required, as no substantial energy penalty is incurred when altering *a*. This procedure accelerates the computation as the majority of the data collection is performed with a fast artificial interaction, but it does not constitute any approximation as it can simply be viewed as establishing a transitional reference system, as is common practice when performing TI.^61,79,80^

The artificial interaction in question is in principle a free choice, but it should be both computationally efficient and close to the physical one to avoid unnecessary computations in the *a*-ensemble. Working with the Coulomb interaction, a variety of cutoff based approximations have been developed, which all could be used as the artificial interaction; see review by Fukuda and Nakamura^81^ and references therein. In this work, we have used the spherically averaged Ewald potential,^82,83^ in particular the formulation by Yakub and Ronchi (YR)^84,85^ that has been successfully applied in MD,^86^ MC^87,88^ and PIMC,^89,90^ and recently has attracted new theoretical interest.^91^ By construction, the YR interaction yields energies similar to the Ewald summation, and its simple algebraic structure makes it cheap to evaluate allowing for classical MC simulation with up to 10^6^ particles.^87^ The new *a*-ensemble with the YR potential as the artificial interaction has been implemented in the ISHTAR code,^92^ which employs the canonical adaptation^93,94^ of the worm algorithm.^95,96^ All reported computations have been performed using the primitive factorization.^33^

Up to this point, only the bosonic sector has been considered to avoid the FSP. To calculate the free energy with Fermi statistics *f*_1_^(F)^ rather than Bose statistics *f*_1_^(B)^ for a system with interaction 1, the sign *S*_1_ in the corresponding system should be resolved:^74^

4 $$f_{1}^{\left(F\right)}-f_{1}^{\left(B\right)}=-\frac{k_{B}T}{N}\;\log\left(S_{1}\right)\equiv \Delta f_{S,1}$$

Equation 4 completes our methodology for free energy computations which is schematically shown in Figure 1 highlighting the steps and Hamiltonians.

**Figure 1:**
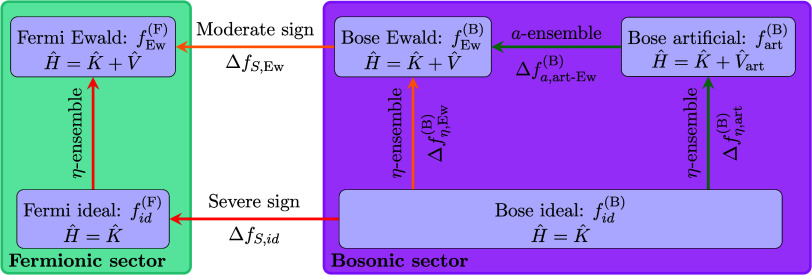
Schematic showing the different systems used to compute the free energies *f* and the Hamiltonians *Ĥ* of each system. Arrows indicate an ensemble or sign computation to go between systems, and labels the associated free energy change *Δf*. Green arrows indicate computations which are computational cheap, while orange arrows indicate moderate cost either due to a costly Ewald computation or the FSP. Red arrows are severely affected by the FSP.

For the sign evaluation in eq 4, the above-mentioned ξ-extrapolation was used based on the functional form:

5 $$S\left(N,\xi \right)=e^{a_{S}\left(N,\xi \right)N\xi }$$

where the primary scaling with *N* and ξ is factored out, and the remaining function *a*_*S*_(*N*, ξ) shows only small deviations from being constant. Dornheim et al. successfully showed that the extrapolation from ξ = – 0.2 based on eq 5 with *a*_*S*_(*N*, ξ) = *a*_*S*_(*N*) is highly accurate for θ = 1.^55^ Figure 2 greatly extends the validation of this extrapolation method by considering a 2 orders of magnitude range for ξ for *N* ≤ 66. Validation of the method to substantially smaller ξ is crucial for modeling larger system sizes, since keeping *ξN* roughly constant maintains a resolvable sign. System sizes up to *N* = 1000 are investigated in Figure 2, and *N* ≥ 264 is needed to converge the finite-size effect to within the statistical error bars. This highlights the need to model large systems to approach the thermodynamic limit.

**Figure 2:**
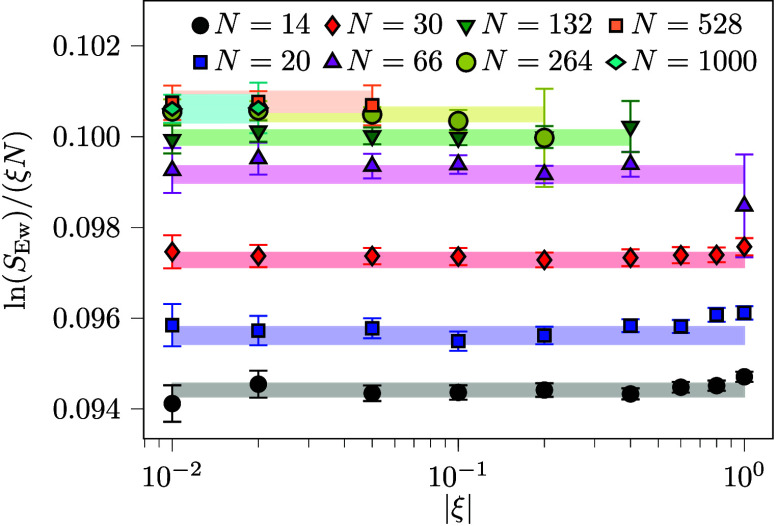
Average sign *S*_Ew_ for the UEG at *r*_*s*_ = 3.23 and θ = 1.0, for different system sizes *N* and permutation weight ξ. Note that ξ < 0 and all values of *S*_Ew_ are less than unity. The error bars, correspond to 95%-confidence intervals estimated from simulations with varying initial conditions. The extrapolation of the confidence interval assuming *a*_*S*_ is independent of ξ is shown in the highlighted areas. The point from which the extrapolation is performed is described in the Supporting Information. Good agreement with the extrapolation is demonstrated, validating the computational model for *S*_Ew_.

The minor systematic error observed in the ξ-extrapolation with *N* = 14 is 0.3% and corresponds to a 0.05 mHa error in free energy. These errors are expected to decrease with the size of the system, where the permutation structure is less affected by boundary effects and self-exchanges;^97^ this makes the generalization of the corresponding free energy difference via the ξ-extrapolation more straightforward. This can be seen particularly well for the more strongly coupled case of *r*_*s*_ = 10 shown in the Supporting Information.

The nonideal contribution to the Fermionic free energy is the exchange correlation free energy:

6 fxc=fEw(F)−fid(F)=Δfη,art(B)+Δfa,art−Ew(B)+(ΔfS,Ew−ΔfS,id)

which in our accelerated scheme (second line) has three distinct contributions. The contribution of the η-ensemble with the artificial interaction *Δf*_*η,art*_^(B)^, the correction from the *a*-ensemble Δ*f*_*a*,art‑Ew_^(B)^ and the difference between the sign contribution for the interacting and noninteracting system Δ*f*_*S*,Ew_ – Δ*f*_*S*,*id*_. In the standard Ewald-only approach, the first two contributions are given by a single term Δ*f*_η,Ew_^(B)^ = Δ*f*_η,art_^(B)^ + Δ*f*_*a*,art‑Ew_^(B)^. The origin of each term is also shown in Figure 1.

As both a conceptual and practical validation of the acceleration method, Figure 3 shows the exchange correlation free energy computed both via the standard Ewald-only method and our accelerated scheme for *N* ≤ 30. The results cannot be distinguished from each other on the scale of Figure 3, and any deviation lies within the statistical error margins. As the system size increases, the accelerated method can perform up to 18 times as many MC steps as the standard method in a given time; see the Supporting Information for additional information. The additional MC samples reduce the statistical error but more crucially allow us to investigate larger system sizes.

**Figure 3:**
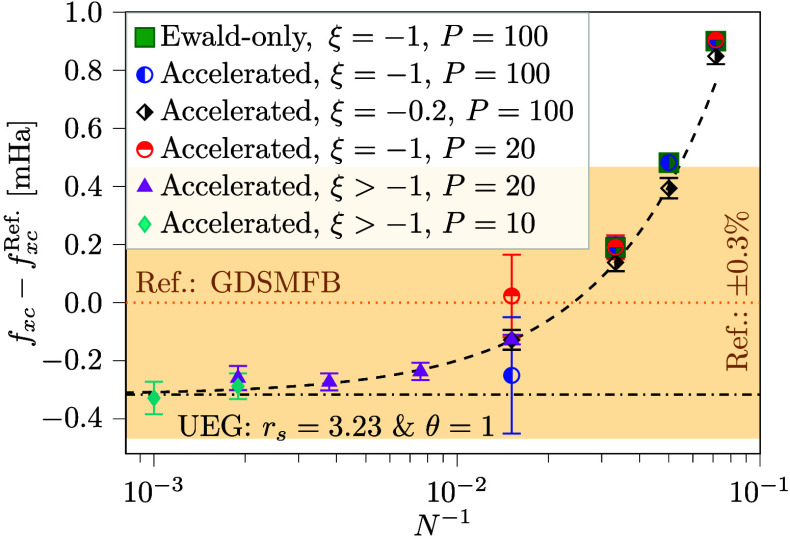
Finite size and finite *P* corrected exchange correlation free energies for the UEG at *r*_*s*_ = 3.23 and θ = 1.0 shown as the difference from the GDSMFB parametrization^98^ with a value of *f*_*xc*_^Ref.^ = −0.15529 Ha (Ref.). The remaining *N* dependence is a fraction of a percent. The computations have been performed in a variety of ways, using the physical interaction (Ewald-only) or accelerated method (Accelerated), with (ξ > −1) and without (ξ = −1) ξ-extrapolation, and varying number of propagators *P*. Overlapping data points are shown when *P* is reduced or extrapolation techniques are employed, demonstrating the correctness of the procedure. The dashed line is a fit on the form *f*_*xc*_(*N*) = *c*_0_ + *c*_1_ *N* ^–*c*_2_^ for *N* ≥ 30, where *c*_0_, *c*_1_ and *c*_2_ are fitting coefficients. The extrapolated free energy is reduced by 0.2% compared to the reference and *c*_2_ ≈ 1.3. Error bars as described in Figure 2.

In Figure 3, the exchange correlation free energy computations are scaled up to 1000 electrons using the accelerated method. To limit computational expense, a reduced number of imaginary time slices *P* is used to factorize the density matrix for large *N*. The finite *P* error has been systematically investigated for smaller *N* with *P*s between 8 and 200 as demonstrated in the Supporting Information. Empirically, we find that the corresponding *P*-correction that connects a finite *P* to the limit of *P* ^–1^ → 0 is independent of *N*, reflecting the local nature of the factorization error, which is ultimately due to the quantum delocalization of individual particles. The correction has been applied in Figure 3. As a further validation of the finite-*P* correction, duplicate data points are shown when *P* is reduced and the results are always within the statistical error.

To further reduce the size dependence of the free energy, the results in Figure 3 have been finite-size corrected using the method introduced by Groth et al.^98^ (see further details in the Supporting Information). The finite-size correction is highly efficient and at the investigated condition removes 93% of the finite-size effect already at the smallest system used, resulting in a remaining finite-size error of the order of 1 mHa per electron. In Figure 3, it is shown that the surviving size-dependent error scales roughly linearly with *N* ^–1^ (dashed black), and for the largest systems investigated, this error is expected to be one hundredth of a mHa. The results are within 0.3% of the GDSMFB parametrization computed by the adiabatic connection formula,^98^ well within the expected error margins of their parametrization. In conclusion, the finite size correction method by Groth et al. is highly efficient and virtually any remaining finite size error has been eliminated by reaching system sizes with 1000 electrons, now numerically feasible with our accelerated technique for free energy calculations.

The magnitude of each of the contributions to the exchange correlation free energy is shown in Figure 4(a). The dominant contribution under the investigated condition is the interaction contribution from the η-ensemble (Δ*f*_η,YR_^(B)^) followed by the sign contribution (Δ*f*_*S*,Ew_ – Δ*f*_*S*,*id*_). The correction for using the artificial interaction in the η-ensemble (Δ*f*_*a*,YR‑Ew_^(B)^) is 3 orders of magnitude smaller than the overall contribution of interactions and vanishes with increasing system size, as the YR potential tend to the Coulomb form. This highlights the efficiency of the YR interaction in mimicking the full Ewald summation with respect to energy, even if some artifacts are present for spatially resolved quantities.^90^ The Δ*f*_*a*,YR‑Ew_^(B)^ correction is small enough for the present system that it could have been neglected for the computations at larger *N*, but the *a*-ensemble will have a substantial contribution for more ordered systems, for other choices of artificial interaction, and for systems with different *V̂*. For the *r*_*s*_ = 10 system, the picture is broadly the same, but the interaction contribution is even more dominant for this strongly interacting case.

**Figure 4:**
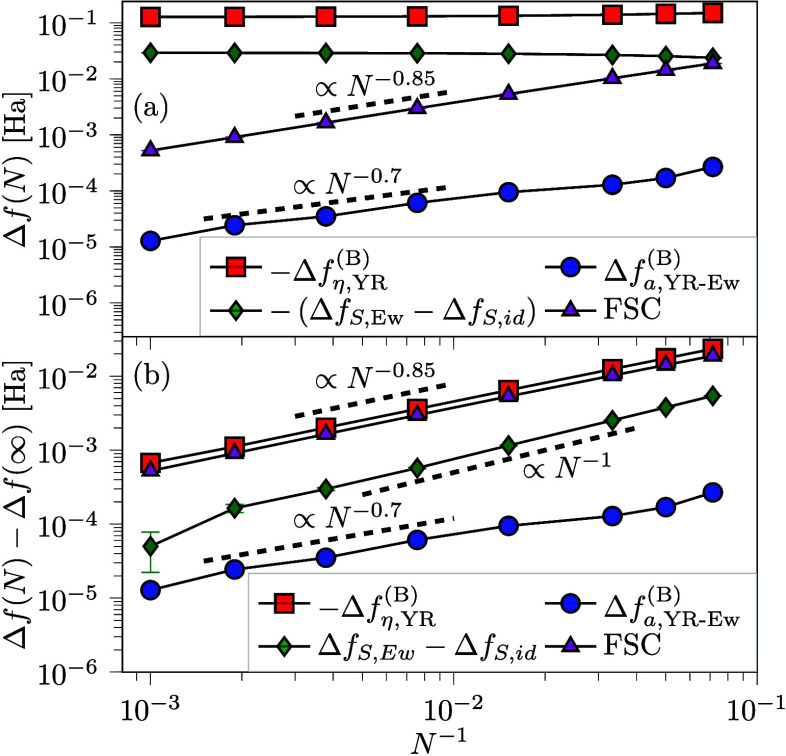
Size (a) and scaling (b) of the *N* dependent free energy for the UEG at *r*_*s*_ = 3.23 and θ = 1.0. The correction term *Δf*_*a,*YR-Ew_^(B)^ and FSC vanish as *N* → ∞ while the contribution from the η-ensemble and the sign converges to a finite value. The subtraction of the infinite system size contribution *N* = ∞ in (b) was facilitated by a fit for *N* ≥ 66 on the form Δ*f* = *d*_0_ + *d*_1_ *N* ^–*d*_2_^, the same functional form used by Demyanov and Levashov.^87^ The interaction components are seen to converge sublinearly (*d*_2_ < 1). Error bars based on statistical error as described in Figure 2.

The approach to the thermodynamic limit for the three contributions to the free energy is highlighted in Figure 4(b) by subtracting the (fitted) thermodynamic limit. The size of the finite *N* errors generally follows the magnitude of each respective term. The finite size error in the sign, which is by far the hardest contribution to compute in practice, is seen to scale linearly with particle number; this might be exploited for further extrapolation and optimization in future works. The two interaction contributions scale sublinearly, with approximate exponents of 0.7 and 0.85, respectively. However, these two exponents are not universal as they increase for the *r*_*s*_ = 10 conditions. In the Supporting Information, the sublinear scaling is discussed in terms of the finite-size correction model. For the simulation with *N* = 1000, all finite-size errors are below 1 mHa and the chemical accuracy is reached even without any finite-size correction procedure.

To summarize, we have introduced and exemplified the use of an accelerated method for free energy estimation based on *ab initio* PIMC. The method accelerates the computation in two primary ways. First, an intermediate “artificial” reference system is introduced in which interactions are numerically evaluated more efficiently. The majority of interaction effects can be captured in this artificial system, and any remaining error can be corrected by the introduced *a*-ensemble which in our work only required a single computation with the numerically more costly physical interaction. In this work, the use of the artificial interaction reduced the computation effort by a factor of up to 18 for the interaction contribution. Second, a ξ-extrapolation methodology is employed to resolve the sign for larger system sizes that are otherwise prevented by the Fermionic sign problem. This extrapolation was shown to be accurate to 0.3% over 2 orders of magnitude in ξ for θ = 1. The generality of the procedure was demonstrated by considering two different density conditions.

Accelerating the calculation of free energies paves the way for scaling up computations to remove the final systematic error – the finite size effects – at warm dense matter conditions. The presented method can be combined with other acceleration techniques to consider even large systems, e.g., employing GPUs,^99^ hierarchical energy evaluation,^100^ and contraction schemes.^101^ High-precision free energy estimates for the UEG open for the possibility to explore a potential spin phase transition at finite temperature, which have been intensely studied in the ground state.^102,103^ Future work might also explore the long-wavelength physics with the presented method via the density stiffness theorem,^104,105^ which relates the static linear and nonlinear density response to free energy differences. In this regard, the simulation of large systems is crucial to study the optical limit of |*k⃗*| → 0, where the minimum wavenumber |*k⃗*| = 2π/*L* is determined by the box length *L*. Lastly, the present study focuses on the UEG, but it is straightforward to apply our methodology to light elements such as hydrogen and beryllium^53^ to inform planetary and inertial confinement fusion modeling. Moreover, our approach can easily be applied to the simulation of inhomogeneous systems such as electrons in a fixed ionic configuration, which might be of great value for the benchmarking of DFT and potentially even for the data-driven construction of novel exchange correlation functionals.^106^

## Supplementary Material





## Notes

Additional details for Monte Carlo updates in the *a*-ensemble, numerical parameters, discussion on acceleration in the η-ensemble, finite size and finite *P* correction procedures, and the case study of the UEG at the density *r*_*s*_ = 10 (PDF)
Transparent Peer Review report available (PDF)
