# Supplementary material for: Accelerated Free Energy Estimation in Ab Initio Path Integral Monte Carlo Simulations
Source: J Phys Chem Lett. 2025 Oct 6;16(41):10639–46. doi: 10.1021/acs.jpclett.5c02193 (PMC12536444; doi:10.1021/acs.jpclett.5c02193)
Supplement: Supplementary file 1 [file jz5c02193_si_001.pdf]

Supporting Information for "Accelerated free energy estimation in *ab initio* path integral Monte Carlo simulations"

Pontus Svensson,[*,†,‡] Fotios Kalkavouras,[¶] Uwe Hernandez Acosta,[†,‡] Zhandos A. Moldabekov,[†,‡] Panagiotis Tolias,[¶] Jan Vorberger,[‡] and Tobias Dornheim[†,‡]

†*Center for Advanced Systems Understanding (CASUS), D-02826 Görlitz, Germany*

‡*Helmholtz-Zentrum Dresden-Rossendorf (HZDR), D-01328 Dresden, Germany*

¶*Space and Plasma Physics, Royal Institute of Technology (KTH), Stockholm, SE-100 44, Sweden*

E-mail: p.svensson@hzdr.de

Extended ensemble and corrections via the a-ensemble

The extended ensemble method[1] is used to calculate the contribution of interactions to the free energy (in the bosonic sector) in two steps. First, the free-energy difference between the interacting and ideal bosonic systems is calculated using the η-ensemble. In particular, for a large number of particles the acceptance probability is reduced for the move which alters η as the likelihood of finding two electrons in close proximity increases in the ideal system, configurations which are strongly suppressed in the interacting one.[2] Therefore, to maintain an ergodic exploration of the extended ensemble, the η-ensemble is subdivided into N_η steps and the free energy difference is computed for a set of η-values, i.e. $\{\eta_i\}_{i=1}^{N_\eta+1}$, where $\eta_1 = 1$, $\eta_{N_\eta+1} = 0$ and $\eta_{i+1} < \eta_i$. To accelerate this process, these computations are carried out with an artificial interaction which mimics our target interaction – the Ewald summation in this case – but is numerically less expensive to evaluate. Here, the spherically averaged Ewald interaction by Yakub and Ronchi (YR)[3,4] was used. The details of the moves in the η-ensemble were given by Dornheim et al..[5] Second, the a-ensembel is used to correct for the use of the artificial interaction in the η-ensemble by considering the Hamiltonian in Equation (3) of the main text. In principle, the a-ensemble can be subdivided into N_a computations with a set of a-values $\{a_i\}_{i=1}^{N_a+1}$, where $a_1 = 1$, $a_{N_\eta+1} = 0$ and $a_{i+1} < a_i$. However, if the artificial interaction is sufficiently similar to the physical one, a single step $N_a = 1$ is sufficient to maintain ergodicity, as has been the case in this work. This is how the method accelerates the computations, as a reduced number of calculations with the more expensive Ewald interactions is needed.

The moves to switch a-values are constructed analogously to the moves in the η-ensemble with the Metropolis-Hastings[6,7] acceptance probabilities

$$A(a_i \to a_{i+1}) = \min\left\{1, \frac{1}{c_{a_i}} \exp\left(\epsilon\left\{(a_i - a_{i+1})V(\boldsymbol{X}) + (a_{i+1} - a_i)V_{\text{art}}(\boldsymbol{X})\right\}\right)\right\}, \tag{S1a}$$

and

$$A(a_{i+1} \to a_i) = \min\left\{1, c_{a_i} \exp\left(\epsilon\left\{(a_{i+1} - a_i)V(\boldsymbol{X}) + (a_i - a_{i+1})V_{\text{art}}(\boldsymbol{X})\right\}\right)\right\}, \tag{S1b}$$

where $\boldsymbol{X}$ is the path configuration, c_{a_i} corresponds to c in Equation (1), $\epsilon = \beta/P$, and P is the number of factorisations of the density matrix. The move only modifies the a-value, and not the path configurations $\boldsymbol{X}$. Furthermore, the move is applied in both the diagonal and off-diagonal sectors of the worm algorithm.[8,9]

The resulting free energy computed from a series of PIMC calculations is $f^{(\text{F})}_{\text{Ew}} = f^{(\text{B})}_{id} + \Delta f^{(\text{B})}_{\eta,\text{Ew}} + \Delta f_{S,\text{Ew}}$ or using the acceleration method $f^{(\text{F})}_{\text{Ew}} = f^{(\text{B})}_{id} + \Delta f^{(\text{B})}_{\eta,\text{art}} + \Delta f^{(\text{B})}_{a,\text{art-Ew}} + \Delta f_{S,\text{Ew}}$, where

$$\Delta f^{(\text{B})}_{\eta,\text{Ew}} = -\frac{1}{\beta N}\sum_{i=1}^{N_\eta} \log\left[\frac{r^{\text{Ew}}_{\eta_i,\eta_{i+1}}}{c_{\eta_i}}\right], \tag{S2a}$$

$$\Delta f^{(\text{B})}_{\eta,\text{art}} = -\frac{1}{\beta N}\sum_{i=1}^{N_\eta} \log\left[\frac{r^{\text{art}}_{\eta_i,\eta_{i+1}}}{c_{\eta_i}}\right], \tag{S2b}$$

$$\Delta f^{(\mathrm{B})}_{a,\text{art-Ew}} = -\frac{1}{\beta N}\sum_{i=1}^{N_a}\log\left[\frac{r^{\text{art-Ew}}_{a_i,a_{i+1}}}{c_{a_i}}\right], \tag{S2c}$$

and

$$\Delta f_{S,\mathrm{Ew}} = -\frac{1}{\beta N}\log\left[S_{\mathrm{Ew}}\right], \tag{S2d}$$

and see Figure 1 in the main text for the origin of each term. In the above, c_{η_i} corresponds to c in Equation (1) in the main text. The ratios of samples in each subsystem in the extended ensemble is $r^{\mathrm{Ew}}_{\eta_i,\eta_{i+1}}$, $r^{\mathrm{art}}_{\eta_i,\eta_{i+1}}$ and $r^{\text{art-Ew}}_{a_i,a_{i+1}}$ in both the η and a-ensembles for the Ewald (Ew) and artificial (art) interactions. The implementation of the η-ensemble was benchmarked by Dornheim et al.[5] and the implementation of the a-ensemble is confirmed by comparing the free energies between the Ewald-only method and our acceleration method. The different schemes agree within the statistical errors; see Figure 3 in the main text.

Numerical parameters for simulations

Some additional numerical parameters used for the PIMC simulations are provided in Table SI, including the ξ-points used for extrapolation and the number of time slices P used to represent the density matrix. In addition, the parameters used for the η-ensemble are shown. The number of subdivisions N_η increases with N, and the η_i grid is nonuniform, as the structural properties of the system change more rapidly with respect to η when approaching the noninteracting limit. This is particularly evident for $N = 1000$ and $r_s = 10.0$ where the last step in η is one hundred times larger than the first, while retaining roughly the same acceptance probability for the η-move. The exact choice of c_η does not influence the result,[2] but for algorithmic efficiency the number of samples in the two partition functions should be approximately equal. Therefore, $\ln c_\eta \approx -\beta N(f_2 - f_1)$, where f_1 and f_2 are the free energy per particle in the two systems in question which are *a priori* unknown. The coefficients c_η in Table SI were obtained by scanning c_η and optimising the acceptance probability. However, the results agree well with the mentioned estimate, even if the free energies are approximated by a classical parametrisation,[10] except for small η where the quantum statistics are more prevalent.

In simulations which utilised the Ewald interaction, the Ewald parameters were optimised such that the energy of the system was converged to six significant digits, using the single image convention in real-space and using a maximal k-vector component of $8\pi/L$ (L is the box length) in reciprocal space.

Computational speedup in η-ensemble

Empirically, we find that the computational cost to perform a Monte Carlo step in ISHTAR – averaged over all types of steps – is approximately:

$$\text{Computational cost}(N,P) = C_0 P + C_1^{\text{Int.}} P N, \tag{S3}$$

Table SI: Summary of computational parameters. The ξ-column describe the ξ value used for the extrapolation in Figures 3 and 4 in the main text, and Figure S4. The P-column describe the number of imaginary time slices used for the computation in Figures 2 and 4 in the main text, and Figure S4. The three final columns gives the details of the η-ensemble computation. Note that an a-ensemble simulation has been performed in each case.

$r_s = 3.23$ & $\theta = 1.0$					
N	ξ	P	N_η	$\{\eta_i\}_{i=1}^{N_\eta+1}$	$\{c_{\eta_i}\}_{i=1}^{N_\eta}$
14	0.2	100	4	{0.0, 0.01, 0.1, 0.5, 1.0}	{1, 5e-1, 7e-3, 10e-4}
20	0.2	100	4	{0.0, 0.01, 0.1, 0.5, 1.0}	{1, 2e-1, 2e-3, 1e-4}
30	0.2	100	4	{0.0, 0.01, 0.1, 0.5, 1.0}	{1, 1e-1, 1e-4, 1e-6}
66	0.2	100	7	{0.0, 0.01, 0.1, 0.2, 0.4, 0.6, 0.8, 1.0}	{1, 2e-1, 3e-2, 2e-4, 6e-5, 1.5e-5, 3e-6}
132	0.1	20	7	{0.0, 0.01, 0.1, 0.2, 0.4, 0.6, 0.8, 1.0}	{1, 1e-1, 1e-2, 1e-7, 8e-10, 2e-10, 1e-11}
264	0.05	20	12	{0.0, 0.01, 0.05, 0.1, 0.2, 0.3, 0.4, 0.5, 0.6, 0.7, 0.8, 0.9, 1.0}	{1, 1, 6e-2, 8e-6, 4e-7, 1e-8, 1e-8, 2e-10, 1e-10, 3e-11, 1e-11, 5e-12}
528	0.02	20	23	{0.0, 0.01, 0.025, 0.05, 0.075, 0.1, 0.15, 0.2, 0.25, 0.3, 0.35, 0.4, 0.45, 0.5, 0.55, 0.6, 0.65, 0.7, 0.75, 0.8, 0.85, 0.9, 0.95, 1.0}	{1, 1, 5e-1, 1e-2, 1e-2, 1e-5, 1e-6, 1e-6, 5e-8, 2e-8, 1e-8, 1e-9, 1e-9, 1e-9, 1e-10, 1e-10, 1e-10, 1e-10, 1e-11, 1e-11, 1e-11, 1e-11, 1e-11}
1000	0.01	10	23	{0.0, 0.01, 0.025, 0.05, 0.075, 0.1, 0.15, 0.2, 0.25, 0.3, 0.35, 0.4, 0.45, 0.5, 0.55, 0.6, 0.65, 0.7, 0.75, 0.8, 0.85, 0.9, 0.95, 1.0}	{1, 1, 1e-1, 2e-3, 1e-3, 1e-9, 1e-11, 2e-13, 1e-14, 1e-15, 1e-16, 1e-17, 1e-17, 1e-18, 1e-18, 1e-19, 1e-19, 1e-20, 1e-20, 1e-20, 1e-21, 1e-21, 1e-21}
$r_s = 10.0$ & $\theta = 1.0$					
N	ξ	P	N_η	$\{\eta_i\}_{i=1}^{N_\eta+1}$	$\{c_{\eta_i}\}_{i=1}^{N_\eta}$
14	0.2	100	5	{0.0, 0.01, 0.1, 0.2, 0.5, 1.0}	{1.0, 1e-1, 1e-2, 1e-6, 1e-11}
30	0.2	100	5	{0.0, 0.01, 0.1, 0.2, 0.5, 1.0}	{1.0, 1e-2, 1e-3, 5e-13, 1e-24}
66	0.2	100	7	{0.0, 0.01, 0.1, 0.2, 0.4, 0.6, 0.8, 1.0}	{1.0, 1e-4, 1e-7, 5e-18, 1e-20, 1e-21, 5e-22}
132	0.1	20	9	{0.0, 0.02, 0.05, 0.1, 0.2, 0.3, 0.4, 0.6, 0.8, 1.0}	{1e-1, 1e-2, 1e-5, 1e-14, 1e-16, 1e-18, 2e-39, 1e-41, 1e-43}
264	0.1	20	11	{0.0, 0.02, 0.05, 0.1, 0.15, 0.2, 0.3, 0.4, 0.5, 0.6, 0.8, 1.0}	{1e-1, 1e-4, 1e-10, 1e-13, 1e-15, 1e-33, 1e-36, 1e-38, 1e-39, 1e-82, 1e-85}
528	0.1	20	16	{0.0, 0.01, 0.02, 0.05, 0.075, 0.1, 0.15, 0.2, 0.25, 0.3, 0.4, 0.5, 0.6, 0.7, 0.8, 0.9, 1.0}	{1, 1e-1, 1e-8, 1e-9, 1e-11, 1e-26, 1e-29, 1e-32, 1e-34, 1e-72, 1e-76, 1e-79, 1e-81, 1e-83, 1e-85, 1e-86}
1000	0.02	20	25	{0.0, 0.001, 0.002, 0.005, 0.01, 0.02, 0.03, 0.05, 0.075, 0.1, 0.125, 0.15, 0.175, 0.2, 0.25, 0.3, 0.35, 0.4, 0.45, 0.5, 0.55, 0.6, 0.7, 0.8, 0.9, 1.0}	{1, 1, 1, 1, 1e-2, 1e-4, 1e-11, 1e-18, 1e-21, 1e-24, 1e-26, 1e-27, 1e-29, 1e-61, 1e-64, 1e-67, 1e-69, 1e-71, 1e-73, 1e-74, 1e-75, 1e-154, 1e-158, 1e-161, 1e-163}

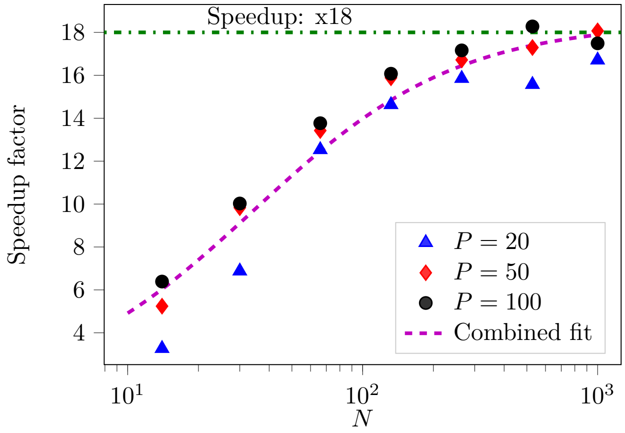

Figure S1: The computational speedup from using the spherically averaged Ewald interaction rather than the full Ewald interaction in the η-ensemble for varying number of P. A fit for the speedup is based on the computational model in Equation (S3), which predicts a maximal speedup of approximately 18 times.

where C_0 refers to computations in the update step and $C_1^{\text{Int.}}$ relates to the computational cost of evaluating the interaction. The latter scales as PN as an order N computation is required to evaluate the potential for each time slice. The speedup in the η-ensemble is achieved as $C_1^{\text{Int.}}$ for the YR interaction is substantially smaller than that for the Ewald interaction.

The computational acceleration was tested by performing computations with both interaction types for a subset of computations needed to evaluate the η-ensemble, and the results are shown in Figure S1. The speedup shows only minor variations with respect to P and tends toward a constant for large N, two properties that are well explained by the model in Equation (S3). For smaller P, numerical overhead not included in Equation (S3) becomes more appreciable. A combined fit over N and P based on the ratio of Equation (S3) for the YR and Ewald interactions is shown to appropriately represent the data. For the larger system sizes investigated, we observe an acceleration of up to 18 times. The exact numerical speedup will depend on the simulation configurations and implementation details, but the results shown here are representative of the computations in the main text.

Finite size corrections

The finite-size corrections (FSC) applied follow the methodology given in the supplementary material of Groth et al..[11] The finite-size error for the exchange correlation free energy at r_s and θ is given by

$$\Delta f_{xc}(r_s, \theta) = \frac{1}{r_s^2} \int_0^{r_s} d\bar{r}_s \, \bar{r}_s \Delta v(\bar{r}_s, \theta), \tag{S4}$$

where $\Delta v(r_s, \theta; N)$ is the finite size error on the interaction energy. The major contribution to Δv is the discretisation error of the interaction integral imposed by the box and not the errors

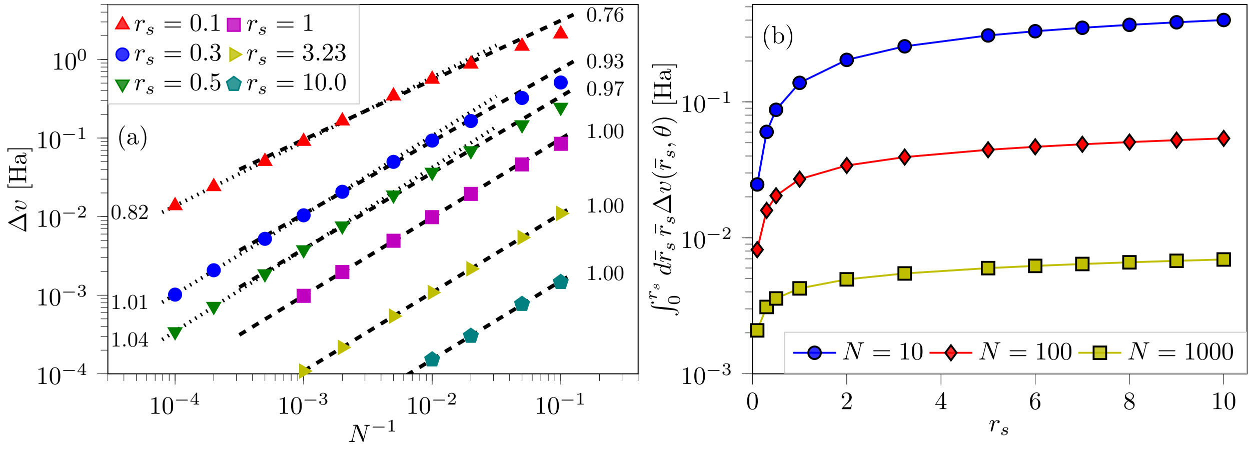

Figure S2: (a) The finite size correction of the interaction energy computed from Equation (S5) at $\theta = 1$ and r_s between 0.1 and 10.0. The lines are scaling fits on the form $\Delta v = v_0 N^{-a}$, where v_0 and a are fitting coefficients. Fits for the intervals $50 \leq N \leq 1000$ (dashed) and $1000 \leq N \leq 10000$ (dotted) are shown with their respective a values to the right and left of the lines, respectively. (b) The integral in Equation (S4) is evaluated for three different particle numbers.

on the structure factor $S(\boldsymbol{k})$ itself.[12] Therefore, the finite-size correction is approximated by

$$\Delta v(r_s, \theta) \approx \frac{1}{2} \int \frac{d\boldsymbol{k}}{(2\pi)^3} \tilde{v}_{\boldsymbol{k}} \left(\bar{S}(\boldsymbol{k}) - 1 \right) - \left(\frac{1}{2L^3} \sum_{\boldsymbol{G} \neq 0} \tilde{v}_{\boldsymbol{G}} \left(\bar{S}(\boldsymbol{G}) - 1 \right) + \frac{\xi_M}{2} \right), \tag{S5}$$

where $\tilde{v}_{\boldsymbol{k}} = 4\pi/\boldsymbol{k}^2$ is the Fourier transformed Coulomb interaction, L is the side length of the box, $\boldsymbol{G} = 2\pi \boldsymbol{n}/L$ where $\boldsymbol{n} \in \mathbb{Z}^3$, and ξ_M is the Madelung constant. As an approximation, the static structure factor is taken from a dielectric theory, commonly the random phase approximation (RPA) $\bar{S}(\boldsymbol{k}) = S_{\text{RPA}}(\boldsymbol{k})$. Malone implemented this procedure in `uegpy`[13] which has been successful in removing most finite-size errors. However, for large numbers of particles this implementation suffers from some stability issues. Therefore, the procedure has been reimplemented with the classical STLS scheme[14] as the underlying dielectric theory, that is, $\bar{S}(\boldsymbol{k}) = S_{\text{STLS}}(\boldsymbol{k})$.

To consider the N-scaling of Δf_{xc}, the scaling of Δv must first be established. For the two conditions under primary investigation here, $r_s = 3.23$ and $r_s = 10.0$, the finite-size error of the interaction energy is seen to scale linearly in Figure S2(a). However, when considering $r_s < 1.0$, we observe a sublinear scaling for particle numbers in the range $50 \leq N \leq 1000$; see Figure S2(a). For a fixed θ, small r_s corresponds to the weak coupling limit as the classical coupling constant $\Gamma_{\text{cl.}}$ which characterises a classical plasma scale as $\Gamma_{\text{cl.}} \propto r_s/\theta$. The Debye length $\lambda_D \propto r_s \Gamma_{\text{cl.}}^{-1/2}$ which is the typical scale length of weakly coupled plasmas grows large compared to the inter-particle separation for small $\Gamma_{\text{cl.}}$, and large numbers of particles must be considered in the modeling. Therefore, we observe an alteration of the N-scaling for $r_s < 1.0$ when considering $N > 1000$. See Caillol and Gilles[15] and Demyanov et al.[16] for further discussion of sublinear scaling in classical MC.

The integral in Equation (S4) accumulates the finite size error of the interaction energy

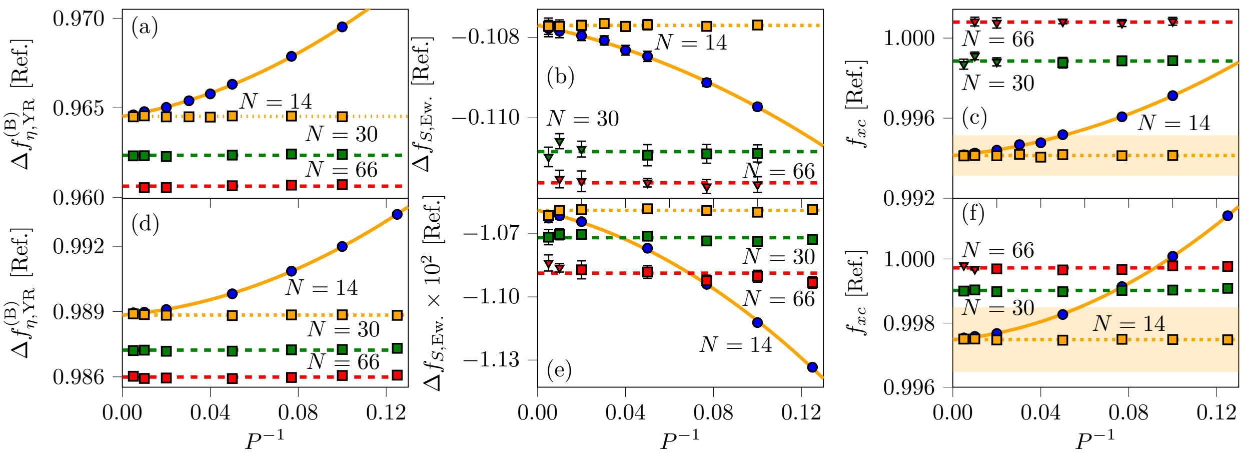

Figure S3: Free energies computed with a varying number of propagators P in units of the GDSMFB parametrisation (Ref.)[11] Results for UEG with $r_s = 3.23$ (top row) and $r_s = 10$ (bottom row), both with $\theta = 1.0$. The finite P error is shown for η-ensemble (left column), sign contribution (middle column) and total exchange-correlation contribution (right column). Uncorrected data (circles) for $N = 14$ are shown along with second-order polynomial fit (solid) and the $P \to \infty$ value (dotted). Corrected data points without (squares) and with (triangles) ξ-extrapolation show only small variations from constants, as indicated by their mean values (dashed). The $N = 30$ and $N = 66$ data in columns 1 and 2 have been shifted vertically, and FSC has been applied to f_{xc}.

for r_s smaller than the target value. As shown in Figure S2(b), a considerable fraction of the integral comes from the region $r_s < 1.0$ where the sublinear scaling is observed for the particle numbers relevant to the main text. In this manner, the sublinear scaling of the energy in the weakly coupled system propagates to the free energy at higher coupling. Within the FSC model and $50 \leq N \leq 1000$, we observe exponents between 0.58 and 0.89 for $\theta = 1.0$ and r_s in the range 0.1 and 10.0. Note that one of the end points of the η-ensemble is the noninteracting limit ($\eta = 0$), and the above reasoning can be translated to the PIMC simulations.

Finite number of propagators: Errors and corrections

The computational cost of PIMC scales linearly with P, and to more efficiently model large N it is desirable to keep P as low as possible. However, a finite P results in a systematic error,[17,18] and convergence must be established. The convergence of $\Delta f^{(\mathrm{B})}_{\eta,\mathrm{YR}}$, $\Delta f_{S,\mathrm{Ew}}$, and f_{xc} for our two test cases with $r_s = 3.23$ and $r_s = 10$, are shown in Figure S3 for $N = 14$. Simulations were performed up to $P = 200$ using the primitive factorisation, and to achieve a systematic error below 0.1% a $P \geq 20$ was required. No substantial P dependence on $\Delta f^{(\mathrm{B})}_{a,\text{art-Ew}}$ was observed.

A very similar systematic trend with P was observed when the above study was carried out for $N = 30$ and $N = 66$, where finite-size errors were seen mainly to shift the result.

Table SII: Coefficients obtained from the fits in Figure S3 using the functional form in Equation (S6). The p_0-coefficients are omitted as they are N dependent. Coefficients are given in units of the GDSMFB parametrisation.[11] Results are shown for $r_s = 3.23$ and $r_s = 10$, both at $\theta = 1.0$ for the UEG.

	$r_s = 3.23$		$r_s = 10.0$	
	p_1 [GDSMFB]	p_2 [GDSMFB]	p_1 [GDSMFB]	p_2 [GDSMFB]
$\Delta f^{(\mathrm{B})}_{\eta,\mathrm{YR}}$	0.021	0.30	0.0086	0.23
$\Delta f_{S,\mathrm{Ew}}$	-0.011	-0.092	-0.0023	-0.030
f_{xc}	0.0100	0.20	0.0060	0.20

Therefore, a second-order polynomial fit of the form

$$f(N, P) = p_0(N) + p_1 P^{-1} + p_2 P^{-2}, \tag{S6}$$

where p_0, p_1 and p_2 are fitting coefficients, were carried out separately for $f = \Delta f^{(\mathrm{B})}_{\eta,\mathrm{YR}}$, $\Delta f_{S,\mathrm{Ew}}$ and f_{xc} for $N = 14$. By subtracting the P-dependence obtained from Equation (S6), virtually all systematic errors are compensated for; see the demonstration in Figure S3. This finite P correction (FPC) method has been applied to all results for both $r_s = 3.23$ and $r_s = 10.0$.

The coefficients for the FPC are shown in Table SII. The quadratic correction dominates, unless $P \lesssim 10$. Furthermore, the finite P error in the η-ensemble is typically larger than for the sign contribution. The coefficients for f_{xc} are approximately the sum of the other two. However, a separate fit has been performed.

Free energy for strongly coupled system: $r_s = 10$ & $\theta = 1.0$

The method presented is not restricted to the conditions discussed in the main text, and as a demonstration of this the corresponding computations for the UEG at $r_s = 10.0$ and $\theta = 1.0$ are shown in Figures S4(a) and S4(b). The structure of the sign extrapolation is generally the same as for $r_s = 3.23$, but a stronger ξ-dependence on a_S is shown particularly for $N = 14$. However, this dependence is completely removed when a system size of $N = 66$ is reached, and above this point all ξ-dependence on a_S can be neglected. This fits well to the heuristic explanation in terms of permutation cycles given in the main text. Compared to the interaction contribution, the sign contribution decreases as r_s increases and a stronger electron coupling is reached. Therefore, statistical errors – primarily from the sign estimation – are less prevalent and the estimates for the (finite-size corrected) free energy are well within 0.05%, see Figure S4(b). The final estimation is 0.01% lower than the GDSMFB parameterisation,[11] which is very accurate within this regime.

Finite P corrected results are shown for $P = 20$ and $P = 100$ in Figure S4(b) with good agreement. However, for the $P = 8$ case, the correction formula in Equation (S6) is not able to fully correct the P-dependent error on the scale of 0.01% and the data points disagree outside the estimate of the statistical error for $N = 264, 528, 1000$. Therefore, there is a lower bound on the needed P to reach a desired accuracy.

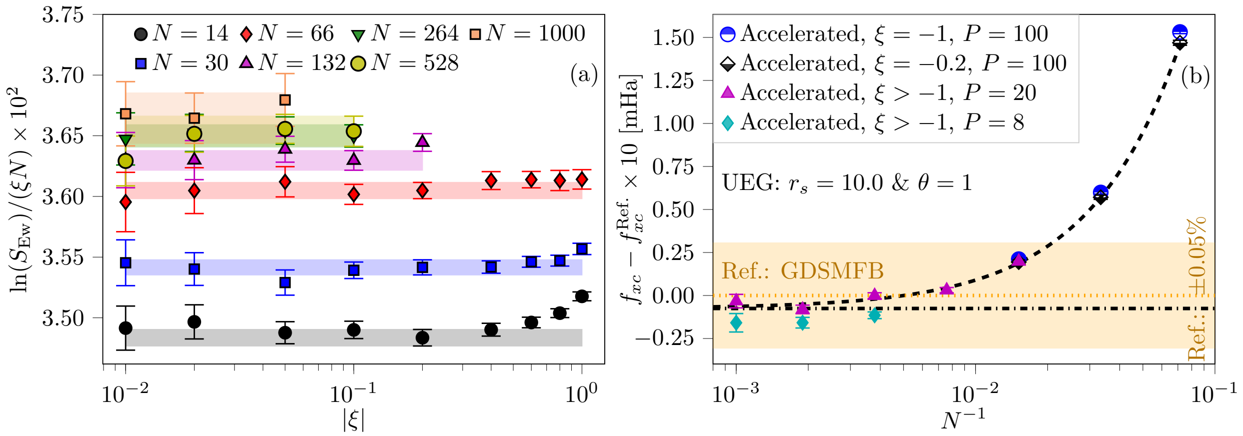

Figure S4: (a) Corresponding figure to Figure 2 in main text for UEG at $r_s = 10$ and $\theta = 1.0$. The extrapolation technique is confirmed at a second condition. Some additional ξ-dependence on a_S is seen for small N, although this is reduced for larger system sizes. (b) Corresponding figure to Figure 3 (main text) for UEG at $r_s = 10$ and $\theta = 1.0$. At these conditions the GDSMFB parametrisation gives $\Delta f_{xc} = -0.061120\,\text{Ha}$ (Ref.). Due to a reduced sign contribution, errors are further reduced compared the corresponding figure in the main text. On the scale of the statistical error, the finite P error can not fully corrected for by the FPC procedure for $P = 8$ as compared to $P = 20$ at $N = 264$.

References

(1) Dornheim, T.; Moldabekov, Z. A.; Schwalbe, S.; Vorberger, J. Direct free energy calculation from *ab initio* path integral Monte Carlo simulations of warm dense matter. *Physical Review B* **2025**, *111*, L041114.

(2) Dornheim, T.; Tolias, P.; Moldabekov, Z. A.; Vorberger, J. η-ensemble path integral Monte Carlo approach to the free energy of the warm dense electron gas and the uniform electron liquid. *Physical Review Research* **2025**, *7*, 023250.

(3) Yakub, E.; Ronchi, C. An efficient method for computation of long-ranged Coulomb forces in computer simulation of ionic fluids. *The Journal of Chemical Physics* **2003**, *119*, 11556–11560.

(4) Yakub, E.; Ronchi, C. A new method for computation of long ranged Coulomb forces in computer simulation of disordered systems. *Journal of Low Temperature Physics* **2005**, *139*, 633–643.

(5) Dornheim, T.; Tolias, P.; Moldabekov, Z.; Vorberger, J. η-ensemble path integral Monte Carlo approach to the free energy of the warm dense electron gas and the uniform electron liquid. *arXiv preprint arXiv:2412.13596* **2024**,

(6) Metropolis, N.; Rosenbluth, A. W.; Rosenbluth, M. N.; Teller, A. H.; Teller, E. Equation of state calculations by fast computing machines. *The Journal of Chemical Physics* **1953**, *21*, 1087–1092.

(7) Hastings, W. K. Monte Carlo sampling methods using Markov chains and their applications. *Biometrika* **1970**, *57*, 97–109.

(8) Boninsegni, M.; Prokof'ev, N.; Svistunov, B. Worm algorithm for continuous-space path integral Monte Carlo simulations. *Physical Review Letters* **2006**, *96*, 070601.

(9) Boninsegni, M.; Prokof'ev, N. V.; Svistunov, B. V. Worm algorithm and diagrammatic Monte Carlo: A new approach to continuous-space path integral Monte Carlo simulations. *Physical Review E* **2006**, *74*, 036701.

(10) Plummer, D.; Svensson, P.; Gericke, D. O.; Hollebon, P.; Vinko, S. M.; Gregori, G. Ionization calculations using classical molecular dynamics. *Physical Review E* **2025**, *111*, 015204.

(11) Groth, S.; Dornheim, T.; Sjostrom, T.; Malone, F. D.; Foulkes, W. M. C.; Bonitz, M. *Ab initio* exchange-correlation free energy of the uniform electron gas at warm dense matter conditions. *Physical Review Letters* **2017**, *119*, 135001.

(12) Dornheim, T.; Groth, S.; Sjostrom, T.; Malone, F. D.; Foulkes, W. M. C.; Bonitz, M. *Ab initio* quantum Monte Carlo simulation of the warm dense electron gas in the thermodynamic limit. *Physical Review Letters* **2016**, *117*, 156403.

(13) See https://github.com/fdmalone/uegpy.

(14) Singwi, K.; Tosi, M.; Land, R.; Sjölander, A. Electron correlations at metallic densities. *Physical Review* **1968**, *176*, 589.

(15) Caillol, J.-M.; Gilles, D. An accurate equation of state for the one-component plasma in the low coupling regime. *Journal of Physics A: Mathematical and Theoretical* **2010**, *43*, 105501.

(16) Demyanov, G.; Onegin, A.; Levashov, P. *N*-convergence in one–component plasma: Comparison of Coulomb, Ewald, and angular–averaged Ewald potentials. *Contributions to Plasma Physics* **2024**, *64*, e202300164.

(17) Sakkos, K.; Casulleras, J.; Boronat, J. High order Chin actions in path integral Monte Carlo. *The Journal of Chemical Physics* **2009**, *130*, 204109.

(18) Dornheim, T.; Groth, S.; Filinov, A.; Bonitz, M. Permutation blocking path integral Monte Carlo: a highly efficient approach to the simulation of strongly degenerate nonideal fermions. *New Journal of Physics* **2015**, *17*, 073017.
