# Supplementary material for: Accelerated Free Energy Estimation in Ab Initio Path Integral Monte Carlo Simulations
Source: J Phys Chem Lett. 2025 Oct 6;16(41):10639–46. doi: 10.1021/acs.jpclett.5c02193 (PMC12536444; doi:10.1021/acs.jpclett.5c02193)
Supplement: Supplementary file 2 [file jz5c02193_si_002.pdf]

jz-2025-021933.R1

Name: Peer Review Information for "Accelerated free energy estimation in <i>ab initio</i> path integral Monte Carlo simulations"

First Round of Reviewer Comments

Reviewer: 1

Comments to the Author

The article is novel and can be published.

Reviewer: 2

Comments to the Author

The authors propose a procedure to speed up path integral Monte Carlo (PIMC) <i>ab initio</i> calculations of free energy for uniform electron gas. The manuscript clearly reflects the complexity of such calculations when dealing with system with fermionic symmetry and also exposes the state-of-art of current strategies to overcome the numerical difficulties. The proposed approach combines previously used method, (a) spherically averaged Ewald interaction as an intermediate system and (b) a ξ-extrapolation technique. Despite my expertise on PIMC calculations for fermionic systems lies on a slightly different context (³He clusters) it becomes clear that the manuscript contains substantial and interesting suggestions which experts in computational physics / chemistry would surely appreciate. It is perhaps its high specificity in numerical and computational issues what might make me wonder whether or not the Journal of Physical Chemistry Letters is the right journal. If editors consider that this does not constitute a problem, I do not see any reason not to suggest its publication.

The only minor things I would point out are:

1.- I find that not eveyone might know what a ξ-extrapolation technique is to be advanced without explanation in the abstract;

2.- The acronyms MC is the only one which is not properly introduced or defined before being used in the text, and in fact it is not used in pg. 5.

3.- The bibliography contains about a 20% of papers by one of the authors. I wonder if that is strictly necessary to conveniently illustrate what is said in the main text.

Reviewer: 3

Comments to the Author

This article presents a very efficient and important methodology for accelerating the estimation of free energy from path integral Monte Carlo simulations by considering an intermediate artificial reference system, where interactions are inexpensive to evaluate numerically. The methodology is based on the spherically averaged Ewald interaction and a ξ-extrapolation technique applied to the uniform electron gas, for which a lot of new and interesting results have been obtained. I would like to see it published in the Journal, but there are a number of concerns that have to be addressed before the publication in order to improve the paper.

In the present work, to overcome the sign problem, the authors use a technique allowing to get information about a fermionic system as an extrapolation of results obtained for a bosonic system.

However, this approach suffers from one physical and one mathematical problem that have to be discussed in detail.

1) From the physical point of view, as it follows from [1] the effective exchange pseudopotential for an ideal fermi system results in repulsion of fermions, while for ideal bosons the effective interaction is attractive. These effective interactions in ideal systems stem from quantum statistics. Surely, strong electron interaction may camouflage these opposite effects for a weak and moderate degeneracy, but this is not the case for strong degeneracy. This physical situation has to be mentioned and discussed in the paper.

1) Huang, K. Statistical Mechanics; John Wiley and Sons: Hoboken, NJ, USA, 1963.

2) From the mathematical point of view to do extrapolation of the thermodynamic functions from bosons to fermions the authors introduced a quantum symmetrized partition function with a formal continuous variable $\xi \in [-1, 1]$. As a result, the new symmetrized partition function depends on the new parameter ξ^{Npp}, where Npp is the number of transpositions in the expansion of each permutation. The authors claim that the factor ξ^{Npp} takes into account the impact of quantum statistics, with $\xi = 1$ corresponding to bosons, $\xi = -1$ to fermions. The function of two variables $f(x, y) = x^y$ at the point (0, 0) has irremovable discontinuity. In fact, along the positive X-axis direction, where y = 0, it is equal to one, and along the positive direction of the Y axis, where x = 0, it is equal to zero. Therefore, the function f(x, y) is non-analytical and its analytical fit numerically obtained for $\xi > 0$ cannot give reliable extrapolation of thermodynamic functions at $\xi < 0$.

Another argument concerning the difficulties of the extrapolation from $\xi = 1$ to $\xi = -1$ was discussed in a recent paper (https://arxiv.org/pdf/2507.22779). The authors of this paper claim that the partition function has (N – 1) zeroes in the range of ξ from -1 to 0. Moreover, the zero at $\xi = -1$ originates an additional term in the free energy, which is absent for bosons. In my opinion, the discussion of these mathematical problems should be added to the paper.

3) On page 2, column 1, last paragraph, it is claimed that “Formally, DFT is exact given the correct exchange-correlation functional…”. This is not true. The Kohn-Sham formulation of DFT containing the exchange-correlation contribution and kinetic energy operator for free particles follows from the Kohn-Sham ansatz, which has not been mathematically proven yet. The mathematically correct exact formulation of DFT was given by M. Levy and E. Lieb.

4) On page 4, column 1, 2nd paragraph, the spherically averaged Ewald potential is assigned to Yakub and Ronchi. In my opinion, this is incorrect. This potential can be found in earlier works [Caillol J.M. J. Chem. Phys. 111, 6528 (1999), formula 2.11], [Angelie A. et al. Monte-Carlo calculations of microfield distributions in a plasma // Colloquium on collisions and radiation. — Orleans, France, 1983. URL: https://inis.iaea.org/api/records/4bsv9-02263/files/15026316.pdf/content]. I think that the idea of angular averaged potential appeared in 1969 in one of CEA-DAM reports. Moreover, Yakub and Ronchi could not prove that practically all coefficients of the Taylor expansion of the potential were zero; they just postulated it. Thus, the contribution of Yakub and Ronchi is not decisive.

5) As the spherically averaged Ewald potential is exact for an unordered system it is unclear why the a-ensemble is necessary. I think that in this case the contribution to Δf would be very small (this is confirmed by figure 4, blue circles). In my opinion, the a-ensemble in the case of spherically averaged Ewald potential can be safely removed from the computational scheme making the calculation even faster.

6) In the caption to Figure 4 there is a fit for Δf in order to calculate Δf(∞). This fit for the calculation of thermodynamic limit was introduced in Ref. 77.

7)The literature review can be extended by several possibilities to overcome the sign problem:

7a) In articles [1,2] to overcome the "sign problem'' the exchange interaction in the density matrix is expressed through a positive semidefinite Gram determinant of the Fermi system, which allows to avoid the sampling of a bosonic partition function for obtaining fermionic expectation values. Due to the Gram determinant, this approach does not suffer from the 'fermionic sign problem'.

1) V. Filinov, P. Levashov and A. Larkin, J. Phys. A Math. Theor. 55 (3), 035001 (2022).

2)V. S. Filinov, R. S. Syrovatka and P. R. Levashov (2022) Solution of the 'sign problem' in the path integral Monte Carlo simulations of strongly correlated Fermi systems: thermodynamic properties of helium-3, Molecular Physics, 120:14, e2102549 DOI: 10.1080/00268976.2022.2102549

7b) An alternative approach based on the Wigner formulation of quantum mechanics in the phase space was used in [3,4] to avoid the antisymmetrization of matrix elements and hence the "sign problem''. This approach allows to reproduce the Pauli blocking of fermions by the effective pair pseudopotential in phase space and it is able to calculate quantum momentum distribution functions, transport properties and average values of quantum operators in the phase space.

3. A.Larkin, V. Filinov, and V. Fortov, Journal of Physics A: Mathematical and Theoretical 51, 035002 (2017)

4. W. Ebeling, V.Fortov, and V. Filinov, Quantum Statistics of Dense Gases and Nonideal Plasmas (Springer, Berlin, 2017).

Reviewer: 4

Comments to the Author

The authors present a method for the acceleration of the calculation of the free energy of a system in path integral Monte Carlo simulations. The proposed method is validated for the spin-unpolarized uniform electron gas. The manuscript is technically sound, its contant is novel, and it certainly deserves publication. However, the main focus of the manuscript must be assigned to the areas of theoretical physics, in particular plasma physics. In my view, other journals with a high reputation and a much stronger focus on physics are more suitable for this manuscript, such as for example Physical Review Letters or Physical Review E.

Author's Response to Peer Review Comments:

Please see the attached response letter and a version of the manuscript (diff.pdf), which highlights the changes made. Changes in the reference list are not directly shown.

Institute CASUS

Helmholtz-Zentrum Dresden-Rossendorf e.V.
Bautzner Landstraße 400 · D-01328 Dresden

Editor of The Journal of Physical Chemistry Letters

Dr. Pontus Svensson
Postdoctoral Researcher
Phone +49 03581-3752351
p.svensson@hzdr.de

September 4th, 2025

Helmholtz-Zentrum Dresden-Rossendorf e. V.

Bautzner Landstraße 400
D-01328 Dresden
www.hzdr.de

Board of Directors:
Prof. Dr. Sebastian M. Schmidt
Dr. Diana Stiller

Company Registration Number:
VR 1693, Amtsgericht Dresden

Bank Details:
Commerzbank AG
IBAN DE42 8508 0000 0402 6573 00
SWIFT DRESDEFF850

VAT-ID-No. DE140213784

Referee response Journal of Physical Chemistry Letters

Dear Editor,

Thank you for sending us the referee comments on our manuscript "Accelerated free energy estimation in *ab initio* path integral Monte Carlo simulations" (ID: jz-2025-021933). We appreciate the time all four referees have dedicated to our manuscript, and are grateful for the positive and constructive feedback. In response, we have mainly expanded the literature review, highlighted general aspects of the methodology, and improved the readability for non-specialist readers. In addition, some requested formatting changes to the reference list have been included.

Please find a copy of the referee report below with our replies interleaved in blue. For the referees' convenience, we have also attached a marked up version of the revised manuscript highlighting all changes.

We hope that, with these changes, the ms will be suitable for publication in the *Journal of Physical Chemistry Letters*.

Yours Sincerely,
Pontus Svensson
(on behalf of all authors)

First referee

Recommendation: This paper represents a significant new contribution and should be published as is.

Comments:
The article is novel and can be published.

We thank the referee for their very positive assessment of our work and appreciate that they recognise the potential of our new method for computing free-energies.

Second referee

Recommendation: This paper is publishable subject to minor revisions noted. Further review is not needed.

Comments:
The authors propose a procedure to speed up path integral Monte Carlo (PIMC) *ab initio* calculations of free energy for uniform electron gas. The manuscript clearly reflects the complexity of such calculations when dealing with system with fermionic symmetry and also exposes the state-of-art of current strategies to overcome the numerical difficulties. The proposed approach combines previously used method, (a) spherically averaged Ewald interaction as an intermediate system and (b) a ξ-extrapolation technique. Despite my expertise on PIMC calculations for fermionic systems lies on a slightly different context (^{3}He clusters) it becomes clear that the manuscript contains substantial and interesting suggestions which experts in computational physics / chemistry would surely appreciate. It is perhaps its high specificity in numerical and computational issues what might make me wonder whether or not the Journal of Physical Chemistry Letters is the right journal. If editors consider that this does not constitute a problem, I do not see any reason not to suggest its publication.

The only minor things I would point out are:

1. I find that not everyone might know what a ξ-extrapolation technique is to be advanced without explanation in the abstract;
   This part of the abstract has been reformulated; now the ξ-extrapolation is first introduced in the main text.

2. The acronyms MC is the only one which is not properly introduced or defined before being used in the text, and in fact it is not used in pg. 5.
   The acronym is now defined on page 3 and used on page 5.

3. The bibliography contains about a 20% of papers by one of the authors. I wonder if that is strictly necessary to conveniently illustrate what is said in the main text.
   We acknowledge the referee's observation that approximately $\sim$ 20% of the cited papers were authored by one of the authors of the present work. It is a consequence of his extensive previous contributions to the fields of both fermionic PIMC and to the study of the warm dense UEG, both of which are highly relevant to the present work. For the sake of completeness, we would thus prefer to keep all relevant citations independent of the involved author(s).

We appreciate the careful examination of the manuscript by the referee and hope that future variants/extensions of the present work will be helpful for the study of ^{3}He clusters, their area of expertise.

Third referee

Recommendation: This paper may be publishable, but major revision is needed; I would like to be invited to review any future revision.

Comments:
This article presents a very efficient and important methodology for accelerating the estimation of free energy from path integral Monte Carlo simulations by considering an intermediate artificial reference system, where interactions are inexpensive to evaluate numerically. The methodology is based on the spherically averaged Ewald interaction and a ξ-extrapolation technique applied to the uniform electron gas, for which a lot of new and interesting results have been obtained. I would like to see it published in the Journal, but there are a number of concerns that have to be addressed before the publication in order to improve the paper.

In the present work, to overcome the sign problem, the authors use a technique allowing to get information

about a fermionic system as an extrapolation of results obtained for a bosonic system. However, this approach suffers from one physical and one mathematical problem that have to be discussed in detail.

1. From the physical point of view, as it follows from [1] the effective exchange pseudopotential for an ideal fermi system results in repulsion of fermions, while for ideal bosons the effective interaction is attractive. These effective interactions in ideal systems stem from quantum statistics. Surely, strong electron interaction may camouflage these opposite effects for a weak and moderate degeneracy, but this is not the case for strong degeneracy. This physical situation has to be mentioned and discussed in the paper.
   1) Huang, K. Statistical Mechanics; John Wiley and Sons: Hoboken, NJ, USA, 1963.
   A comment along these lines has been added when the ξ-extrapolation is introduced. As we explain in the manuscript, the method is indeed limited to weak/moderate degeneracy, where the discussed difference between the Bose and Fermi systems is not dominant, see e.g., Dornheim *et al.* (J. Chem. Phys. **159**, 164113, 2023). For systems with a large electrostatic coupling, interaction effects can dominate over statistical ones and further justify the extrapolation. This is indirectly seen in the $r_s = 10$ case presented in the supplementary information, where the statistical contribution $\Delta f_{S,\mathrm{Ew}}$ is small compared to the interaction contribution $\Delta f^{(\mathrm{B})}_{\eta,\mathrm{Ew}}$.

2. [Author's note: In the forwarding of this comment, the $\hat{}$ symbols seem to have been lost. They have been included below for clarity.] From the mathematical point of view to do extrapolation of the thermodynamic functions from bosons to fermions the authors introduced a quantum symmetrized partition function with a formal continuous variable $\xi \in [-1, 1]$. As a result, the new symmetrized partition function depends on the new parameter $\xi^{N_{pp}}$, where N_{pp} is the number of transpositions in the expansion of each permutation. The authors claim that the factor N_{pp} takes into account the impact of quantum statistics, with $\xi = 1$ corresponding to bosons, $\xi = -1$ to fermions. The function of two variables $f(x, y) = x^y$ at the point $(0, 0)$ has irremovable discontinuity. In fact, along the positive X-axis direction, where $y = 0$, it is equal to one, and along the positive direction of the Y axis, where $x = 0$, it is equal to zero. Therefore, the function $f(x, y)$ is non-analytical and its analytical fit numerically obtained for $\xi > 0$ cannot give reliable extrapolation of thermodynamic functions at $\xi < 0$.
   The ξ-dependent factor $\xi^{N_{pp}}$ appears in a sum over permutations. As the number of transpositions N_{pp} for each permutation is a **non-negative integer**, the full partition function can be written as a polynomial in ξ (see also the paper discussed below). For the identity permutation, we define $\xi^0 \equiv 1$ for all ξ, and the boltzmannonic contribution from distinguishable particles never depends on ξ. However, the other permutation sectors do depend on $\xi^{N_{pp}}$, where $N_{pp} \geq 1$, and their contribution vanishes in the Boltzmann case of $\xi = 0$. In this formulation, there is no discontinuity preventing the extrapolation. A slight reformulation has been made in the text.

   Another argument concerning the difficulties of the extrapolation from $\xi = 1$ to $\xi = -1$ was discussed in a recent paper (https://arxiv.org/pdf/2507.22779). The authors of this paper claim that the partition function has (N – 1) zeroes in the range of ξ from -1 to 0. Moreover, the zero at $\xi = -1$ originates an additional term in the free energy, which is absent for bosons. In my opinion, the discussion of these mathematical problems should be added to the paper.
   The archive paper in question only directly discusses zeros in the ground state (the finite T case they claim will be analyzed in a as of yet not available follow-up). A challenging ξ structure at $T = 0$ is not surprising; it is well known that the sign vanishes for $\beta \to \infty$, and fermionic PIMC becomes ill-defined. However, there is no reason to expect such difficulties would persist at finite temperatures, and we cite multiple works evaluating various observables in the range $\xi \in [-1, 1]$ without observing a corresponding pole for moderate degeneracy. A short remark about the ground state discussion has been added to the manuscript.

3. On page 2, column 1, last paragraph, it is claimed that "Formally, DFT is exact given the correct exchange-correlation functional...". This is not true. The Kohn-Sham formulation of DFT containing

the exchange-correlation contribution and kinetic energy operator for free particles follows from the Kohn-Sham ansatz, which has not been mathematically proven yet. The mathematically correct exact formulation of DFT was given by M. Levy and E. Lieb.

Admittedly, the strategy of using PIMC to improve the exchange-correlation discussed here is primarily geared towards the Kohn-Sham formulation, but the comment was made concerning the original DFT formulations provided by Hohenberg and Kohn (1964) and Mermin (1965). We thank the referee for pointing out the formal developments regarding representability of ρ by Levy (1979) and Lieb (1983). As the "exactness" of DFT is not central to the current manuscript, the relevant discussion has been reformulated to avoid this phrasing.

4. On page 4, column 1, 2nd paragraph, the spherically averaged Ewald potential is assigned to Yakub and Ronchi. In my opinion, this is incorrect. This potential can be found in earlier works [Caillol J.M. J. Chem. Phys. 111, 6528 (1999), formula 2.11], [Angelie A. et al. Monte-Carlo calculations of microfield distributions in a plasma // Colloquium on collisions and radiation. — Orleans, France, 1983. URL: https://inis.iaea.org/api/records/4bsv9-02263/files/15026316.pdf/content]. I think that the idea of angular averaged potential appeared in 1969 in one of CEA-DAM reports. Moreover, Yakub and Ronchi could not prove that practically all coefficients of the Taylor expansion of the potential were zero; they just postulated it. Thus, the contribution of Yakub and Ronchi is not decisive.

   The two additional references are now cited, and the wording has been altered. If the referee has a more concrete reference for the 1969 report in question (which could not be located), we are happy to include this one as well. A specific reference to Yakub and Ronchi is still needed due to variations in the definition for the potential offset. Regarding the coefficients of the Taylor expansion, this was discussed by Demyanov and Levashov (J. Phys. A: Math. Theor. **55** 385202, 2022). However, for the current work, this is of secondary importance as any reasonable approximation for the Ewald potential will suffice.

5. As the spherically averaged Ewald potential is exact for an unordered system it is unclear why the a-ensemble is necessary. I think that in this case the contribution to Δf would be very small (this is confirmed by figure 4, blue circles). In my opinion, the a-ensemble in the case of spherically averaged Ewald potential can be safely removed from the computational scheme making the calculation even faster.

   The contribution from the a-ensemble is indeed small and even below 1 mHa for $r_s = 3.23$ computations. The contribution has been included for completeness and to make the computations in the Bose sector approximation free. For the current scenario, it could have been neglected, but this might not be true for other interactions and/or choices of artificial interaction. A comment regarding this has been included in the manuscript. With regards to efficiency, the a-ensemble is the least costly contribution to evaluate, and only constitutes a moderate cost for completeness.

6. In the caption to Figure 4 there is a fit for Δf in order to calculate $\Delta f(\infty)$. This fit for the calculation of thermodynamic limit was introduced in Ref. 77.

   The fitting form is a very general one and was largely motivated by the observation of the sublinear scaling. Ref. 77 was cited in the supplementary material with regard to this sublinear scaling, but is now also included in the caption of Figure 4.

7. The literature review can be extended by several possibilities to overcome the sign problem:

   a. In articles [1,2] to overcome the ''sign problem'' the exchange interaction in the density matrix is expressed through a positive semidefinite Gram determinant of the Fermi system, which allows to avoid the sampling of a bosonic partition function for obtaining fermionic expectation values. Due to the Gram determinant, this approach does not suffer from the 'fermionic sign problem'.

      1) V. Filinov, P. Levashov and A. Larkin, J. Phys. A Math. Theor. 55 (3), 035001 (2022).

2) V. S. Filinov, R. S. Syrovatka and P. R. Levashov (2022) Solution of the 'sign problem' in the path integral Monte Carlo simulations of strongly correlated Fermi systems: thermodynamic properties of helium-3, Molecular Physics, 120:14, e2102549 DOI: 10.1080/00268976.2022.2102549

b. An alternative approach based on the Wigner formulation of quantum mechanics in the phase space was used in [3,4] to avoid the antisymmetrization of matrix elements and hence the "sign problem". This approach allows to reproduce the Pauli blocking of fermions by the effective pair pseudopotential in phase space and it is able to calculate quantum momentum distribution functions, transport properties and average values of quantum operators in the phase space.
3. A.Larkin, V. Filinov, and V. Fortov, Journal of Physics A: Mathematical and Theoretical 51, 035002 (2017)
4. W. Ebeling, V.Fortov, and V. Filinov, Quantum Statistics of Dense Gases and Nonideal Plasmas (Springer, Berlin, 2017).

The introductory discussion on the sign problem has been extended. Additional methods to those suggested have been included.

We thank the third referee for their detailed comments; addressing these has certainly improved the manuscript as a whole.

Fourth referee

Recommendation: While the work is good and publishable, a more appropriate journal is recommended such as Physical Review Letters

Comments:
The authors present a method for the acceleration of the calculation of the free energy of a system in path integral Monte Carlo simulations. The proposed method is validated for the spin-unpolarized uniform electron gas. The manuscript is technically sound, its contant is novel, and it certainly deserves publication. However, the main focus of the manuscript must be assigned to the areas of theoretical physics, in particular plasma physics. In my view, other journals with a high reputation and a much stronger focus on physics are more suitable for this manuscript, such as for example Physical Review Letters or Physical Review E.

We appreciate the positive comments made by the fourth referee and thank them for their report. Regarding the choice of journal. We believe that the Journal of Physical Chemistry Letters is well-suited for the manuscript as it is aimed at ***physical chemists***, *biophysical chemists,* ***chemical physicists***, ***physicists***, ***material scientists***, *and engineers*; we believe that the current work is relevant to at least four of these groups (in bold font). The strong overlap between the physics and chemistry communities on the topic is further attested by the considerable number of references we make to articles published in, e.g., *The Journal of Chemical Physics* (11), *Journal of Chemical Theory and Computation* (1) and, indeed, JPCL (2) itself. Therefore, we are convinced that JPCL will be the optimal venue for our work to reach both physicists and chemists, and will facilitate a broader impact than a more specialised physics journal.

Formatting requests

1. References 23, 47, 61, 80, 82 are incomplete:
*Please include author names, article titles, journal name, publication year, and at least the first page number for each reference citation for the following incomplete journal references: 61, 82.
References 61 and 82 has been extended.
*Please include author names, title of article, name of repository (section name, if provided), submission date, DOI or URL (accessed YYYY-MM-DD) for the following incomplete preprint

references: 23, 47, 80 ...etc.
References 23 and 80 has been extended. Reference 47 has been replaced by journal reference.
